# Suppression of the Testis-Specific Transcription of the ZBTB32 and ZNF473 Genes in Germ Cell Tumors

**DOI:** 10.32607/actanaturae.11620

**Published:** 2022

**Authors:** S. S. Bulanenkova, O. B. Filyukova, E. V. Snezhkov, S. B. Akopov, L. G. Nikolaev

**Affiliations:** Shemyakin-Ovchinnikov Institute of Bioorganic Chemistry, Russian Academy of Sciences, Moscow, 117997 Russia

**Keywords:** zinc-finger proteins, testis, germ cell tumors, transcription

## Abstract

The family of genes containing C2H2 zinc finger domains, which has more than 700 members, is one of the largest in the genome. Of particular interest are C2H2 genes with potential tissue-specific transcription, which determine the functional properties of individual cell types, including those associated with pathological processes. The aim of this work was to identify C2H2 family genes with tissue-specific transcription and analyze changes in their activity during tumor progression. To search for these genes, we used four databases containing data on gene transcription in human tissues obtained by RNA-Seq analysis. The analysis showed that, although the major part of the C2H2 family genes is transcribed in virtually all tissues, a group of genes has tissue-specific transcription, with most of the transcripts being found in the testis. After having compared all four databases, we identified nine such genes. The testis-specific transcription was confirmed for two of them, namely ZBTB32 and ZNF473, using quantitative PCR of cDNA samples from different organs. A decrease in ZBTB32 and ZNF473 transcription levels was demonstrated in germ cell tumors. The studied genes can serve as candidate markers in germ cell tumors.

## INTRODUCTION

The family of genes containing zinc-finger domains of the C2H2 type includes more than 700 members and is one of the most numerous [1, 2]. One of the main functions of the zinc finger structure is DNA binding; therefore, many proteins of this family have the properties of transcription factors [1]. Apart from zinc fingers, different family members may contain additional N-terminal domains, such as KRAB, SCAN, and BTB/POZ, which determine the regulatory functions of proteins [3]. Despite the evolutionary prevalence of this family in mammals [4], its significance for cell activity is still not entirely clear to us. In general, studies are focused on a detailed analysis of the structure and function of individual family members such as CTCF [5, 6]. Some genes are associated with a series of diseases [7]. Considering the large number of family members, attempts are being made to define more general and universal functions for its members. For instance, their involvement in the organization of the chromatin structure is considered [8]. In particular, their ability to ensure the proximity of distant genomic regions through zinc fingers binding to DNA and protein–protein contacts is assumed [5]. One of the functions of the protein family containing the KRAB repressor domain is to suppress the activity of retroelements, whose large number explains the wide distribution of the C2H2 family in the human genome [9, 10]. An additional difficulty in the study of this family is introduced by the fact that the properties of zinc fingers are not limited to DNA binding; a number of these domains also interact with RNA and proteins [7, 11, 12]. Thus, the study of this family seems to be a massive and multi-stage endeavor aimed at finding out whether different family members are related to each other through a common functional unity and whether they perform narrow specific functions or act as multifunctional proteins.

Taking into account the diversity of the members of the family, it is sensible to assume their involvement in the regulation of various biological processes, both those common to all cells and those specific to individual cell types. A natural question arises about the activity of the C2H2 family members in various pathologies, including malignant cell transformation. Taking into account the fact that the number of tissue-specific genes is an order of magnitude lower than that of ubiquitously expressed genes and that most of them are expressed in the testes [13, 14], it is safe to assume that this pattern is also preserved among C2H2 family genes.

In this work, we analyzed several databases containing information on gene transcription in different human body tissues and identified several testis-specific genes of the C2H2 family. We experimentally confirmed the highly specific transcription of two of these genes in the testes compared to other tissues. Analysis of tumor and normal testicular tissues showed suppressed gene transcription in germ cell tumors.

The study of testis-specific genes might eventually help us to better understand the processes of tumorigenesis and their possible practical application in predicting, diagnosing, and treating cancer.

## EXPERIMENTAL

**Data sources**

Averaged data on gene transcription levels in different tissues presented in TPM (Transcripts Per Million, [15]) were obtained from https://proteinatlas.org (Human Protein Atlas, HPA), https://gtexportal.org/ home/ (The Genotype-Tissue Expression, GTEX, version GTEx_Analysis_2017-06-05_v8_RNASeQCv1.1.9_ gene_median_tpm.gct.gz, without transformed lymphocytes), https://www.ebi.ac.uk/ (using data from E-MTAB-513 (Illumina Body Map), E-MTAB-4344 (ENCODE project), E-MTAB-2836 (HPA), and E-MTAB-5214 (GTEx)). Complete data on gene transcription in all tissue samples presented in TPM were obtained from the GTEx website (https://gtexportal. org/home/), version GTEx_Analysis_2017-06-05_v8_ RNASeQCv1.1.9_gene_tpm.gct.gz.

Tissue-specific genes were selected for each database as described below. First, all genes with an expression level of at least 5 TPM were selected from each tissue. Further analysis was carried out for each selected gene. The gene expression level in a particular tissue was compared with that in other tissues. The ratio of the gene expression level in TPM to its expression levels in other tissues was calculated for each tissue. Next, the minimum value was chosen from the set of values obtained for each tissue. If this value was at least 3, the gene was assumed to possess tissue-specific expression.

**Tissue samples**

Lung, kidney, large and small intestine, skeletal muscle, lymph node, spleen, and anterior cerebral cortex samples were obtained from healthy adult patients who had died from injuries incompatible with life. Testicular tumor samples (31) were obtained by orchiectomy; they included 27 samples of germ cell origin and 11 samples of adjacent normal tissues, with 7 germ cell samples (testicular parenchyma) among them. A total of 18 samples were paired (6 pairs of germ cell origin and three pairs of non-germ cell origin). Two samples of normal testicular tissue, obtained during surgical castration of patients with prostate cancer, were further used as controls (see supplementary Table_S1 for details; available upon request). All representative samples were immediately frozen in liquid nitrogen. The samples were collected according to Federal Law No. 180 “On Biomedical Cell Products” (Order of the Ministry of Health of the Russian Federation No. 517n dated August 11, 2017; Appendix 2, see http://publication.pravo.gov.ru/Document/ View/0001201709290030) and approved by the ethical committees of the Institute of Bioorganic Chemistry n.a. M.M. Shemyakin and Yu.A. Ovchinnikov of the Russian Academy of Sciences and N.N. Blokhin National Medical Research Center of Oncology of the Ministry of Health of the Russian Federation.

**RNA isolation**

Total RNA was isolated using guanidine isothiocyanate according to [16]. All RNA preparations were treated with DNase I (Promega, USA) according to the manufacturer’s recommendations. Final samples were purified using the RNeasy MINI RNA kit (Qiagen, USA). The quality and purity of the RNA samples were determined by electrophoresis in 1% agarose gel. RNA concentrations were determined by spectrophotometry.

**Quantitative PCR of cDNA (RT-PCR)**

The first strands were synthesized using a random hexanucleotide primer (Promega) and PowerScript reverse transcriptase (Clontech, USA) according to the manufacturer’s instructions. The cDNA template amount in each PCR reaction was equivalent to 10 ng of total RNA. The sequences of the primers used in the study are presented in Table 1. Primers were selected using the Primer–Blast software (https://www.ncbi.nlm.nih.gov/tools/primer-blast/index.cgi), with primer location in different exons being one of the criteria. The reaction was performed in the qPCR-HS SYBR buffer system (Evrogen, Russia) on a LightCycler 480 PCR platform (Roche, USA) using the following temperature program: 95°C for 3 min, followed by 40 cycles at 95°C for 20 s, 65°C for 20 s, and 72°C for 40 s. The transcription level was assessed relative to the geometric mean abundance of the 18S rRNA and GAPDH transcripts. All experiments were performed in three technical replicates.

**Statistical analysis of experimental data**

Comparison of two data groups was performed using the Mann–Whitney test for independent samples and the Wilcoxon test for paired samples. In order to analyze the transcription consistency, the Spearman correlation coefficient was calculated. The significance level was set at 0.05. All database calculations were performed using Excel2010, the R software environment [17], and the stats and openxlsx (https://github. com/ycphs/openxlsx) packages. To determine the tissue specificity of gene transcription, the ratio of the transcription level for each gene expressed in TPM in all tissues, except for the studied one, to the gene transcription in the studied tissue was calculated; this made it possible to avoid division-by-zero errors in the absence of transcription in other organs. The highest ratio was determined for each gene; if it did not exceed 0.3, then the gene was selected for further analysis.

**Table 1:** Sequences of primers for quantitative PCR

Gene	Forward	Reverse	Product length, bp
18S	TGAGAAACGGCTACCACATC	GCTATTGGAGCTGGAATTACC	203
GAPDH	ACTCCTCCACCTTTGACGCT	TCTTCCTCTTGTGCTCTTGCT	179
ZBTB32	GCCCTATGCGTGCTCTGTCT	GGTCATGGCCGAGAAGTCC	139
ZNF473	GGAAGCCCAGAAGCAACAAG	TTCTGGATCGCCTAGCAAACT	189
ZNF446	AATAGAGGGGTCTGTCCAGC	CCGTACTTCTCCAGCATCGC	231

Correlation matrices were constructed using the stats, cluster (https://CRAN.R-project.org/package=cluster), and corrplot (https://github.com/taiyun/corrplot) packages. For cluster analysis, dendrogram construction, and calculation of the optimal number of clusters, we used the stats, cluster, dendextend [18], NbClust [19], and clValid [20] packages. A value equal to 1 - Spearman’s rank correlation coefficient modulus was used as a measure of the distance between genes. Clusters were defined using the hierarchical classification algorithm and the complete linkage method. Scripts are available upon request.

**Additional Online Resources**

Generation of Venn diagrams: http://bioinformatics. psb.ugent.be/webtools/Venn/;

GePIA (Gene Expression Profiling Interactive Analysis, http://gepia.cancer-pku.cn/) [21]: comparison of gene transcription levels in normal and tumor samples based on GTEx (https://gtexportal.org/home/) and TCGA (The Cancer Genome Atlas Program, https:// portal.gdc.cancer.gov/) data;

HGNC (HUGO Gene Nomenclature Committee): https://www.genenames.org/;

Ensembl: https://www.ensembl.org/index.html;

Online Gene ID to Gene Symbol Converter: https:// www.biotools.fr/;

Human genome browser [22]: https://genome.ucsc. edu/

## RESULTS

**Nine genes of the C2H2 family have testis-specific transcription**

Large-scale RNA sequencing data provided by the Illumina Body Map, Encyclopedia of DNA Elements (ENCODE) [23], Genotype-Tissue Expression (GTEx) [24], and Human Protein Atlas (HPA) [25] projects were used for the analysis. The presented data sources differ in the number of tissues and samples per tissue (ranging from one to several hundreds) and the method of biomaterial sampling used (collection during either surgery or autopsy). A more detailed description of databases is presented in [13].

We analyzed the transcription levels averaged over several samples and presented in TPM. C2H2 family genes were selected from each data set; the selection was based on the fact of belonging to group 28 in the HGNC database [26], using Ensembl identification numbers. The number of tissues and genes of the C2H2 family, presented in different databases, is shown in Table 2.

**Table 2:** Representation of tissues and genes in databases

Database	Tissues, number	C2H2 gene, number
Illumina Body Map	16	717
ENCODE	13	718
GTEx	53	718
HPA	43	709

Next, we estimated the total number of genes of this family transcribed in different tissues at a level of at least 3 TPM. Except for a few tissues where the number of genes transcribed at this level is in the range of 200–300 (e.g., liver and skeletal muscle), most tissues contain more than 450 such genes, with the largest number of genes (over 600) transcribed at this level in the testes. Thus, we can conclude that most of the genes of this family are active in almost all the presented tissues (see supplementary Table_S2; available upon request).

Next, we searched for the C2H2 family genes specific to each tissue. First, we selected genes with a transcription level of at least 5 TPM in a specific tissue. A gene was considered active predominantly in a given tissue if its transcription level was at least three times higher than that in any other tissue or organ.

The results of the analysis of the tissue-specific transcription of the genes presented in each of the databases are shown in supplementary Table_S3 (available upon request). We would like to note that all four databases provide data only for tissues of the adrenal glands, testicles, ovaries, liver, and lung. In all cases, the largest number of genes with tissue-specific transcription (≥ 16) was found in the testes. C2H2 genes with tissue-specific transcription (≤ 10) were also found in ovaries, brain, spleen, cerebral cortex, bone marrow, and prostate. However, unlike in the case of testes, the comparison of the results presented in different databases revealed almost no common genes. This observation is consistent with the results of [14], which revealed 35 genes with tissue-specific transcription, while the number of testis-specific genes of the C2H2 family in other organs and tissues did not exceed six. Thus, while a major portion of C2H2 family genes are non-specifically transcribed in most tissues, the largest tissue-specific fraction of the genes is transcribed in the testes.

Further, we focused on this gene fraction. A total of 52 such genes were found in all the databases. It should be noted that the number and set of genes vary slightly between different database versions. This is due to the fact that, when new data are added to the databases, the average transcription level changes and the genes at the boundaries of the conditions set may end up on either side. It is also worth mentioning that databases such as Illumina and Encode include a small number of tissues (and the smallest number of samples per tissue: from one to three) compared to HPA and GTEx, which increases the probability of a false identification of a gene as testis-specific, due to the lack of information on its transcription in tissues not present in the database. Therefore, we further analyzed 25 genes whose transcription levels in the testes exceeded the maximum level in other organs by more than 1.5-fold in all considered databases. Of these, 13 genes coincided with the data from [14] on 35 testis-specific genes. The discrepancies can be explained by the use of different databases (GTEx and HPA in this study and TiGER in [14]) and the different algorithms used to search for tissue-specific genes. Thus, it can be seen that gene selection depends on the search algorithm and the database version. All of this points to the great importance of directly confirming the analyzed data experimentally.

At the same time, there are differences in the transcription profiles of the selected genes. CTCFL, PRDM9, and ZNF560 are highly testis-specific genes; their transcription levels in other tissues do not exceed 0.6 TPM. Furthermore, the transcription level of these genes in the testes is approximately 2–3 times lower than that of the ZBTB32, ZNF165, ZNF473, and ZNF541 genes. The levels of ZBTB32, and ZNF541 are almost undetectable in most tissues (the median varies in the range of 0–0.6 TPM). However, ZBTB32 transcription can reach 7.5 TPM in a number of organs and cells, such as B cells, lymph node, appendix, spleen, tonsils, and Peyer’s patches, while the ZNF541 gene level in the adrenal glands reaches 2–3 TPM. ZNF165, ZSCAN5A, ZNF487, and ZNF473 are transcribed at a low level in almost all the tissues, and their level is at least three times higher in the testes than in any other tissue.

**Fig. 1:**
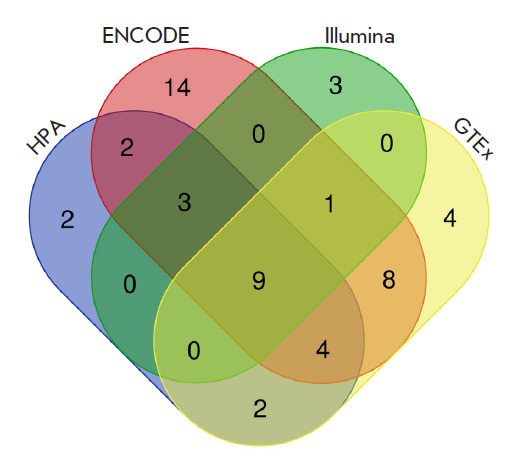
Venn diagram showing preselected genes common to the four databases: Human Protein Atlas, ENCODE, Illumina BodyMap, and GTEx. The diagram was generated using the online program (http://bioinformatics.psb.ugent.be/webtools/Venn/)

We selected the genes common to all databases from the sets of genes isolated from each database (Fig. 1). A total of nine genes were selected: ZBTB32, CTCFL, ZNF560, ZNF541, ZNF473, ZNF165, PRDM9, ZSCAN5A, and ZNF487. All these genes, with the exception of PRDM9, ZSCAN5A, and ZNF487, are defined as testis-specific by using various database versions and are also present in the group of testis-specific genes [14]. Data on the transcription of nine genes in the testes according to the four analyzed databases, as well as the median and maximum values of gene transcription in tissues other than the testes, are presented in Table 3; the structural characteristics of the genes are shown in Table 4 (similar data for all 52 genes are provided in supplementary Table_S4; available upon request).

At the same time, there are differences in the transcription profiles of the selected genes. CTCFL, PRDM9, and ZNF560 are highly testis-specific genes; their transcription levels in other tissues do not exceed 0.6 TPM. Furthermore, the transcription level of these genes in the testes is approximately 2–3 times lower than that of the ZBTB32, ZNF165, ZNF473, and ZNF541 genes. The levels of ZBTB32, and ZNF541 are almost undetectable in most tissues (the median varies in the range of 0–0.6 TPM). However, ZBTB32 transcription can reach 7.5 TPM in a number of organs and cells, such as B cells, lymph node, appendix, spleen, tonsils, and Peyer’s patches, while the ZNF541 gene level in the adrenal glands reaches 2–3 TPM. ZNF165, ZSCAN5A, ZNF487, and ZNF473 are transcribed at a low level in almost all the tissues, and their level is at least three times higher in the testes than in any other tissue.

For further analysis, we selected two genes, ZNF473 and ZBTB32, which are important for determining the tissue identity of the testes [27].

**Transcription of ZBTB32 and ZNF473 is suppressed in testicular germ cell tumors**

**Fig. 2:**
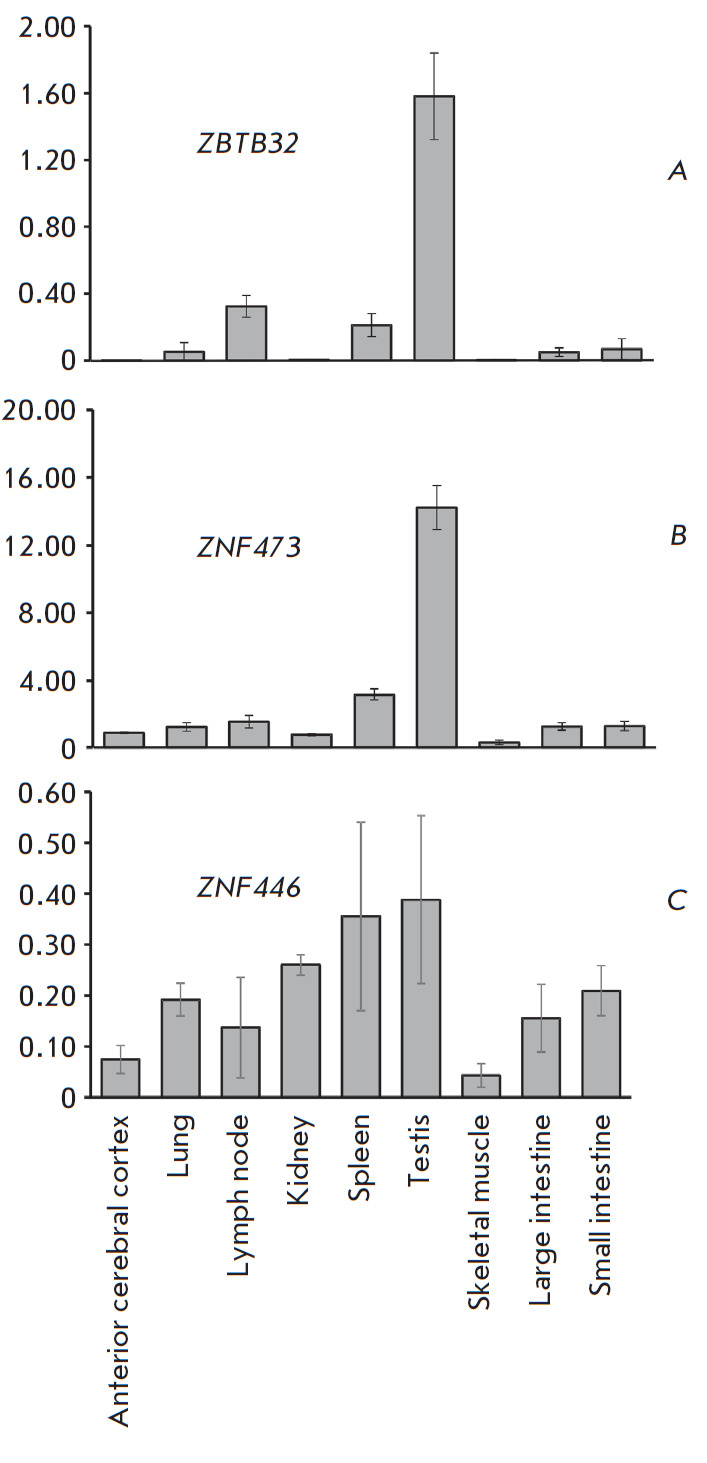
Relative content of *ZBTB32 *(*A*)*, ZNF473 *(*B*)*, *and *ZNF446 *(*C*) transcripts in various tissues. The RNA level was normalized to the geometric mean levels of the housekeeping 18S rRNA and *GAPDH *gene transcripts

We experimentally determined the levels of the ZBTB32 and ZNF473 transcripts in samples of human testes, lung, kidney, large and small intestine, skeletal muscle, lymph node, spleen, and anterior cerebral cortex. The transcription level was assessed by real-time PCR using a cDNA template and geometric mean levels of GAPDH and 18S rRNA transcripts for normalization. The results are presented in Fig. 2A,B. As it can be seen from the figure, the ZNF473 and ZBTB32 levels in the testes exceed those in other organs by at least five- and four-fold, respectively. Low but detectable levels of ZNF473 and ZBTB32 were observed in lymphoid tissues (spleen and lymph nodes), while being insignificant in the other tested tissues, which is consistent with the results of the database analysis discussed above.

**Table 3:** Transcription of selected genes in testes according to four databases (in TPM)*

Gene	HPA	ENCODE	GTEx	Illumina
ZNF473	76.4 (11.1/4.25)	79 (8/2.5)	49.1 (8.3/3.25)	46 (8/4)
ZBTB32	43.3 (7.5/0.15)	84 (4/0)	109.3 (6.5/0.3)	32 (5/0.4)
ZNF541	19.2 (1.7/0.1)	40 (1/0.3)	45.7 (2.7/0.3)	18 (2/0.3)
ZSCAN5A	34.6 (11.3/3.4)	12 (2/0.95)	14.4 (2.3/1.2)	12 (3/1)
ZNF487	51.4 (9.9/2.95)	28 (7/2)	23.2 (4.4/2.1)	21 (5/2)
PRDM9	7 (1.7/0)	9 (0/0)	6.9 (0/0)	6 (0/0)
ZNF560	11.9 (1.2/0)	15 (0.2/0)	15.5 (0.6/0)	12 (0.3/0)
CTCFL	20.4 (0.8/0.3)	14 (0.2/0)	7.5 (0.1/0)	17 (0.5/0,2)
ZNF165	35.8 (7.4/1.55)	46 (15/2)	45.6 (7.6/1.35)	49 (9/2)

^*^Maximum and median transcription values for selected genes in other tissues (in TPM) are presented according to the same database and indicated in brackets with a slash.

In order to compare the transcription profiles of the two studied genes and confirm their independence from the aspects of sample preparation, reaction conditions, etc., we performed a parallel transcriptional analysis of a randomly selected gene, ZNF446, which also belongs to the C2H2 family. This gene was not selected from the databases according to the abovementioned criteria; it transcribed in testes and other tissues at a low level (1–10 TPM), without a pronounced tissue specificity. Our results confirm the absence of a tissue-specific transcription of ZNF446 (Fig. 2C).

**Table 4:** Data on protein domains and gene location

Gene C2H2	number	Other	domains Location
CTCFL	11	–	20q13.31
PRDM9	14	SET, KRAB	5p14.2
ZBTB32	3	BTB/POZ	19q13.12
ZNF165	6	SCAN	6p22.1
ZNF473	20	KRAB	19q13.33
ZNF541	5	ELM2, SANT	19q13.33
ZNF560	15	KRAB+KRAB	19p13.2
ZSCAN5A	5	SCAN	19q13.43
ZNF487	3	KRAB	10q11.21

**Fig. 3:**
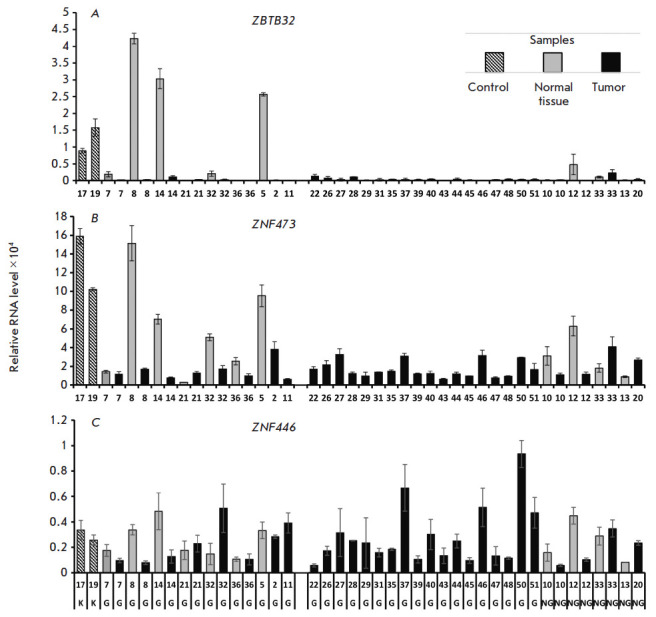
Relative levels of *ZBTB32 *(*A*)*, ZNF473 *(*B*)*, *and *ZNF446 *(*C*) transcripts in testicular tumor germ and non-germ cell samples. Control samples (healthy testicular parenchyma) are indicated by the dashed line, samples from healthy tissue adjacent to the tumor are highlighted in gray, and tumor tissue samples are marked in black. Healthy and tumor tissue samples sharing the same number belong to the same patient. The letters at the bottom stand for: K – control samples; G – germ cell tumor samples; NG – tumor samples that do not contain germ cells. The RNA level was normalized to the geometric mean levels of 18S rRNA and *GAPDH
*

We determined the transcription levels of the same genes using a cDNA panel obtained from tumor and normal testicular tissues. Transcription levels were assessed as described above. The results are shown in Fig. 3. The panel is represented by parenchyma samples from healthy testes (control samples 17N and 19N), tumors, and normal tissues adjacent to them. A number of samples are represented by tumor/adjacent conditionally normal tissue (normal) pairs obtained from one patient. Tumors (and adjacent norms) are represented by both samples of germ cell origin (seminoma, teratoma, yolk sac tumor, embryonic cancer, and mixed tumors) and non-germ cell samples, represented by the stromal and paratesticular tumors (Leydig cell tumor, rhabdomyosarcoma, and leiomyosarcoma).

In germ-cell tumors, ZBTB32 transcription is suppressed to an almost undetectable level (Fig. 3A), while ZNF473 transcription is decreased to the values characteristic of other tissues (Fig. 3B). No clear patterns of changes in the ZNF446 transcription were found during tumor formation (Fig. 3C).

The samples of normal tissues adjacent to germ cell tumors are characterized by a large spread in the ZBTB32 and ZNF473 transcription levels from values close to those in the control samples (samples 5N, 8N, and 14N) to ones characteristic of tumors (samples 7N and 21N). In paired samples with a high level of normal transcription (pairs No. 8, 14, and 32), ZBTB32 and ZNF473 transcription in the tumor is downregulated at least eight and three times, respectively. It should be noted that ZNF446 transcription can be either down- or upregulated in these samples. One of the reasons for the spread in the ZBTB32 and ZNF473 transcription levels in the samples adjacent to a germ cell tumor may be the onset of malignant cell transformation in tissues that are morphologically defined as normal. In addition, an effect of certain tumor cell types on neighboring tissues cannot be excluded. For example, transcription of testis-specific genes of the PIWI family in tumor-adjacent tissue was shown to be associated with the tumor type [28]. However, no patterns were noted between the tumor type and gene transcription level in the adjacent normal tissue in the studied samples. The study of the effect of different tumors on the properties of adjacent tissues may be a promising task for future research.

There is a spread in the expression levels of ZNF473, ZBTB32, and ZNF446 in non-germ cell tissues. Comparison of the transcript levels of these genes in non-germ tumors (samples 10T, 12T, 20T, and 33T) and adjacent normal tissues (samples 10N, 12N, 13N, and 33N) reveals multidirectional changes in the expression of all three genes during the formation of non-germ tumors and no clear patterns of changes in the transcription levels of all three genes.

In general, a significant decrease in the ZNF473 and ZBTB32 transcription levels is observed in tumor as compared to healthy tissue (the Mann–Whitney p-value is < 0.02 in both cases and >0.4 in ZNF446). These genes can act as markers for germ cell-derived tumors.

To confirm these data, we compared the transcription levels of 25 previously identified genes in normal and tumor testis samples (Table 5) using the online resource GePIA [21]. The transcription level of most of the genes (with the exception of ZNF728, ZNF560, and ZFP42) in the tumor is reduced by more than three-fold, to values comparable to those in other tissues. The transcription level of ZNF728 in the tumor decreases by less than two-fold, the level of ZNF560 remains almost unchanged, while the level of ZFP42, on the contrary, increases by 17 times.

The results we obtained for the ZBTB32 and ZNF473 genes are consistent with the GePIA data. This allows us to consider other genes with similar behavior as potential markers of tumor formation in the testes, according to the GePIA. One can assume that most of the studied genes are part of the networks of intergenic interactions responsible for the main cellular processes determining testis functions; these processes are suppressed in tumorigenesis.

**Fig. 4:**
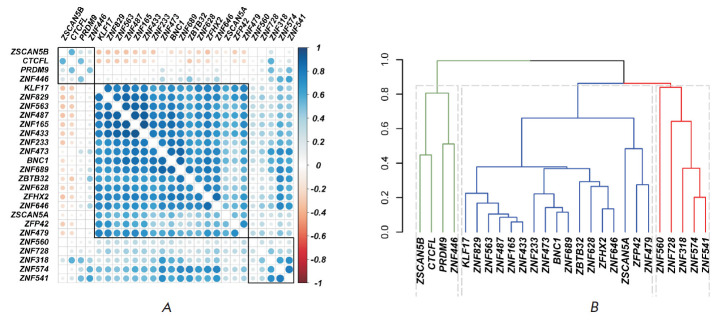
Correlation matrix of the transcription levels of 25 genes selected based on their testis-specific expression and the control gene *ZNF446 *in 361 testis samples from the GTEx collection (*A*). The genes are arranged according to hierarchical clustering using the complete linkage method. Cluster boundaries are outlined in black. Spearman’s correlation coefficients are presented; their values are indicated by color. Dendrogram shows the consistency of gene expression (*B*). As a measure of the difference, a value equal to 1 - the Spearman correlation coefficient modulus was used. Three clusters are outlined; they are indicated by different colors; the dotted gray line indicates cluster boundaries

In this regard, the similarity of the transcription profiles for ZBTB32 and ZNF473 in germline samples between normal and tumor tissue is important; it suggests the consistency of their transcription. We analyzed the transcriptional consistency of 25 selected testis-specific genes using the GTEx database containing data of large-scale RNA sequencing in 361 normal testis samples and generated a correlation matrix for them and the control ZNF446 gene (Fig. 4A).

Using correlation coefficients as a measure of the distance between the genes according to the formula (1 - the correlation coefficient modulus), we created a hierarchical tree using the hierarchical classification algorithm (Fig. 4B). It should be noted that, when using this approach, the most “closely located” genes are the ones with an increased correlation coefficient, regardless of its sign. The optimal number of clusters is within the range of 2–3, as determined by different algorithms. There is a good correlation between different methods of hierarchical classification (correlation coefficients for cophenetic analysis exceed 0.51). When clustered by different methods, most of the genes, including ZNF473 and ZBTB32, fall into one cluster, while only four genes, namely CTCFL, ZSCAN5B, PRDM9, and the control gene ZNF446 falls into another cluster. Thus, most of the selected genes are consistently transcribed in the testes. The transcription levels of the genes within the same cluster are positively correlated with each other. The genes included in different clusters can be part of different branches of the gene network specific to testes and, consequently, be involved in different biological processes in testes. Establishing the position of the studied genes in the hierarchy of intergenic interactions and their relationship with intracellular processes in the testis is a massive, but promising, undertaking for future research.

## DISCUSSION

The study of gene expression is of particular interest in the case of pathological processes, including malignant cell transformation. An important step is the search for genes that can be further used as diagnostic markers or objects for targeted gene therapy. Of particular interest are genes with pronounced tissue-specific expression, since they provide a specific cellular response to external and internal stimuli. In this work, we chose the family of genes encoding C2H2 zinc finger domains as the study object. This family is of particular interest, because, firstly, most of its members, due to the presence of a DNA- binding domain, belong to transcription factors: i.e., regulatory genes, and, secondly, the large size of the family makes it likely to identify a number of peculiar patterns in their gene expression.

**Table 5:** Transcription levels of testis-specific C2H2 genes in normal and tumor testis samples according to GePIA*

Gene	Normal, TPM	Tumor, TPM	Normal/tumor ratio, times	p-value
ZBTB32	93.0	0.93	100.0	1.78e–77
PRDM9	4.52	0.05	90.4	7.58e–78
ZNF541	27.4	0.36	76.2	1.19e–99
KLF17	15.8	0.31	51.0	5.38e–24
CTCFL	9.11	0.20	45.6	1.60e–77
ZNF479	3.34	0,13	25.7	6.61e–39
ZFHX2	17.7	1.09	16.2	4.19e–56
ZNF487	33.0	2.48	13.3	4.01e–63
ZNF433	32.9	2.61	12.6	1.77e–75
ZSCAN5B	2.25	0.18	12.5	7.78e–45
ZNF165	21.3	2.20	9.68	2.25e–44
ZNF563	15.3	1.98	7.71	2.93e–70
ZNF473	39.1	6.37	6.14	3.95e–47
ZSCAN5A	34.6	5.94	5.83	1.09e–59
ZNF628	31.7	5.45	5.82	7.35e–45
ZNF233	9.55	1.68	5.68	1.84e–32
ZNF829	12.6	3.28	3.85	5.86e–20
ZNF646	32.7	8.83	3.70	3.21e–38
ZNF689	22.4	6.40	3.50	1.54e–33
ZNF318	46.8	14.3	3.27	7.26e–34
BNC1	19.6	6.45	3.04	3.99e–9
ZNF574	37.0	15.7	2.36	2.47e–22
ZNF728	5.86	3.44	1.70	1.94e–7
ZNF560	8.89	7.53	1.18	4.09e–2
ZFP42	2.54	43.0	0.06	2.86e–34

^*^Transcription medians are shown in TPM. Genes are arranged in decreasing order of the ratio of their transcription levels in normal tissue and tumor. Nine genes selected simultaneously from four databases are highlighted in bold.

To date, several large databases on gene expression in various tissues and organs have been created thanks to developments in large-scale sequencing technology. It is possible to select candidate genes using user-defined algorithms to search for genes with tissue-specific expression. Databases differ in the number of and method used to obtain samples; therefore, it is important to correctly compare the obtained results when searching for and analyzing candidate genes. In this work, we performed a simultaneous analysis of four databases based on the average level of gene expression in a tissue/organ in each database. As a result, nine genes of the C_2_H_2_ family with potential testis-specific transcription were selected. Two genes, ZBTB32 and ZNF473, were chosen for further analysis; their tissue-specific transcription in testicular parenchyma cells has been confirmed experimentally

Important parameters of gene expression include its change during malignant transformation of cells. The online resource GePIA (Gene Expression Profiling Interactive Analysis) is dedicated to this type of data. The resource is based on an algorithm that allows one to compare large-scale sequencing data obtained from two sources: the GTEx collection of normal tissues and the TCGA collection of tumor tissues. According to the latter resource, transcription of ZBTB32, ZNF473, PRDM9, CTCFL, ZNF165, ZNF541, as well as a number of other genes, is downregulated in testicular germ cell tumors. Therefore, these genes can be considered as potential markers of malignant transformation of germ cells. We have experimentally confirmed a decrease in the transcription level of two selected genes (ZBTB32 and ZNF473) in germ cell tumors. No clear patterns in the expression of these genes in non-germ cell tumors and adjacent normal tissues were found. The expression of these genes in normal non-germ cell tissues is initially low, and it remains at this level in non-germ cell tumors. A decrease in the transcription of these genes in germ cells can serve as a risk marker for the development of germ cell tumors. The absence of ZBTB32 transcription can also serve as evidence of a lack of contamination of adjacent normal tissues when obtaining a tumor sample in experiments in which the purity of the tumor sample is important. However, in the latter case, additional markers are necessary to distinguish germ cell tumors from non-germ cells.

Thus, the data on gene expression accumulated in databases is of great help in the search for candidate genes that could be involved in pathological processes. Further analysis in the form of experimental confirmation of the patterns revealed in silico, the identification of gene functions, and position in the hierarchy of gene networks is an interesting but massive task for future research.
